# ﻿Two new species of *Phylloporia* (Hymenochaetales) from the Neotropics

**DOI:** 10.3897/mycokeys.90.84767

**Published:** 2022-06-07

**Authors:** Meng Zhou, Fang Wu, Yu-Cheng Dai, Josef Vlasák

**Affiliations:** 1 Institute of Microbiology, School of Ecology and Nature Conservation, Beijing Forestry University, Beijing 100083, China Beijing Forestry University Beijing China; 2 Biology Centre of the Academy of Sciences of the Czech Republic, Branišovská 31, CZ-370 05 České Budějovice, Czech Republic Biology Centre of the Academy of Sciences of the Czech Republic České Budějovice Czech Republic

**Keywords:** Hymenochaetaceae, n28S, phylogeny, taxonomy

## Abstract

Two new species of *Phylloporia*, *P.crystallina* and *P.sumacoensis*, are described based on 28S ribosomal RNA phylogeny, morphology, host, and geographic distribution. *Phylloporiacrystallina* is characterized by pileate, perennial basidiomata with a duplex context, small pores 9–10 per mm, a monomitic hyphal system, absence of cystidia and cystidioles, presence of large rhomboid crystals in tube trama, broadly ellipsoid to subglobose basidiospores measuring 2.8–3 × 2–2.3 μm, and growth on angiosperm stump. *Phylloporiasumacoensis* is characterized by pileate, perennial basidiomata with a duplex context, very small pores 10–12 per mm, a monomitic hyphal system, hyphae at dissepiment edges bearing fine crystals, presence of cystidioles, broadly ellipsoid to subglobose basidiospores measuring 3–3.7 × 2.1–2.8 μm, and growth on living liana.

## ﻿Introduction

*Phylloporia* Murrill (Hymenochaetaceae, Hymenochaetales) was established with *P.parasitica* Murrill as the type (Murrill 1904). The genus is characterized by annual or perennial, effused-reflexed, pileate or stipitate, soft corky to hard corky basidiomata, tomentose to velutinate pileal surface, a context mostly duplex with a black line between upper tomentum and lower contextual layer, a monomitic hyphal system in most species, generative hyphae with simple septa, absence of setal elements (with the exception of *Phylloporiamori* Wu et al.), and subglobose, ellipsoid or cylindric, hyaline to yellowish, fairly thick-walled basidiospores which are usually collapsed when mature and < 6 µm in the greatest dimension. *Phylloporia* species mostly grow parasitically on living angiosperm trees, causing a white rot. Phylogenetically, *Phylloporia* is related to *Flaviporellus* Murrill and *Fulvifomes* Murrill, but *Flaviporellus* and *Fulvifomes* have mostly homogeneous contexts (Wu et al. 2022).

Seventy-one species are currently recognized in *Phylloporia*, among them 17 and 37 species from the Neotropics and China, respectively (Wu et al. 2022). Because more tree species occur in Neotropics than in China (Anonymous 1997) and species diversity of *Phylloporia* is related to tree species diversity (Wu et al. 2019), it seems probable that many unknown species of *Phylloporia* exist in the Neotropics. During investigations of the neotropical polypores, specimens morphologically corresponding to *Phylloporia* were collected from Ecuador. Based on morphological, ecological, and phylogenetic evidence, we hereby propose two new species of *Phylloporia*.

## ﻿Materials and methods

Studied specimens are deposited in herbaria of the Institute of Microbiology, Beijing Forestry University (BJFC) and the National Museum Prague of Czech Republic (PRM). The sections were prepared in 5% potassium hydroxide (KOH), Melzer’s reagent (IKI), and Cotton Blue (CB). The following abbreviations are used: **KOH** = 5% potassium hydroxide, **IKI** = Melzer’s reagent, **IKI–** = neither amyloid nor dextrinoid, **CB** = Cotton Blue, **CB–** = acyanophilous, **CB (+)** = cyanophilous after 12 hours stained with Cotton Blue, L = mean spore length (arithmetic average of spores), **W** = mean spore width (arithmetic average of spores), **Q** = variation in the ratios of L/W between specimens studied, and n = number of spores measured from given number of specimens. The microscopic procedure follows Dai (2010), and the special color terms follow Petersen (1996) and Anonymous (1969). Sections were studied at magnifications up to 1000× using a Nikon Eclipse 80i microscope with phase contrast illumination. Drawings were made with the aid of a drawing tube. Microscopic features, measurements, and illustrations were made from the slide preparations stained with Cotton Blue. Microscopic measurements were made from slide preparations stained with Cotton Blue.

**Figure 1.**
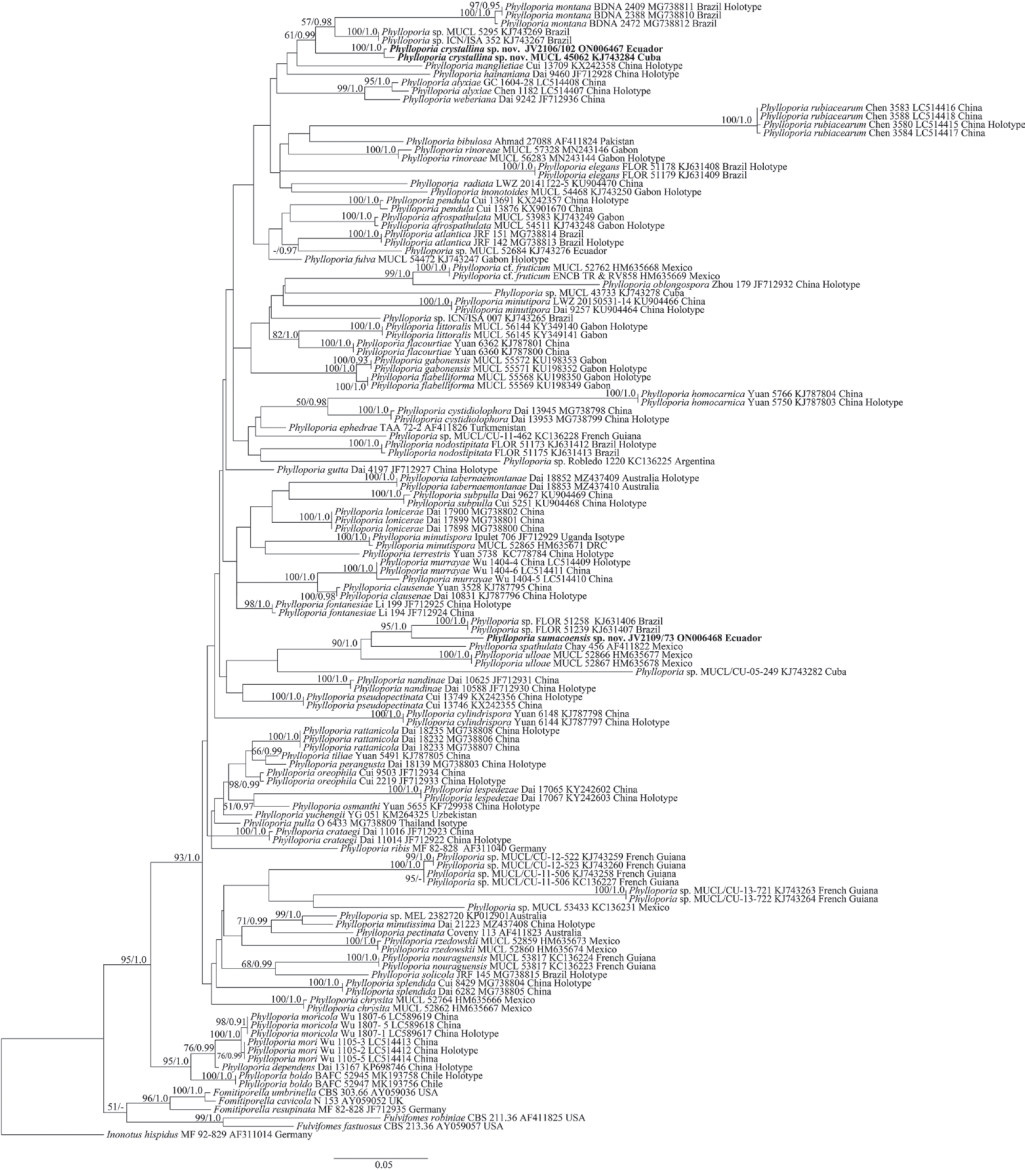
Phylogeny of *Phylloporia* inferred from the 28S dataset. The topology is from the ML analysis, and the support values from ML (former) and BI (latter) analyses simultaneously greater than or equal to 50% and 0.90 are indicated at the nodes, respectively. The new species are in boldface.

The extraction of total genomic DNA from frozen specimens followed Góes-Neto et al. (2005) using the protocol of CTAB 2%. The CTAB rapid plant genome extraction kit-DN14 (Aidlab Biotechnologies Co., Ltd, Beijing) was used to obtain PCR products from dried specimens, following the manufacturer’s instructions with some modifications (Chen et al. 2015, 2016). The primer pairs LR0R and LR7 (Vilgalys and Hester 1990) and LR0R and LR5 (White et al. 1990) were used for PCR amplification. The PCR procedure for 28S was as follows: initial denaturation at 94 °C for 1 min, followed by 35 cycles at 94 °C for 30 s, 50 °C for 1 min and 72 °C for 1.5 min, and a final extension of 72 °C for 10 min. The PCR products were purified and directly sequenced at Beijing Genomics Institute. All newly generated sequences were deposited at GenBank (Sayers et al. 2022) and are shown in the tree (Fig. 1).

In addition to the newly generated sequences, 28S sequences (Fig. 1) from Zhou (2016), Ren and Wu (2017), Qin et al. (2018), and Wu et al. (2019, 2022) were downloaded from GenBank and included in the dataset for phylogenetic analysis. *Inonotushispidus* (Bull.) P. Karst. was selected as outgroup following Zhou (2016). The dataset was aligned using MAFFT v. 7.0 with the Q-INS-i strategy with default parameters (Katoh et al. 2019), and then edited as necessary in BioEdit v. 7.0.5.3 (Hall 1999). Sequence alignments were deposited at TreeBase (submission ID: 29564). Maximum Likelihood (ML) and Bayesian Inference (BI) methods were used for the phylogenetic analysis. The GTR+I+ G model was estimated as the best-fit evolutionary model by MrModeltest v. 2.3 (Nylander 2004) using the Akaike information criterion (AIC). The ML analysis was carried out with raxmlGUI v. 1.2 (Stamatakis 2006; Silvestro and Michalak 2012), and the BI tree reconstruction was carried out with MrBayes v. 3.2.5 (Ronquist et al. 2012). Four Markov chains were run for two runs from random starting trees for 10 million generations, and trees were sampled every 100 generations. BI analysis stopped after effective sample sizes (ESSs) reached more than 200 and the potential scale reduction factors (PSRFs) were close to 1.000 for all parameters. Branches that received bootstrap support for ML (BS) and Bayesian Posterior Probability (BPP) methods greater than or equal to 75% (BS) and 0.95 (BPP) were considered as significantly supported, respectively.

## ﻿Results

### ﻿Phylogenetic results

Two 28S sequences were generated in this study and were deposited in GenBank. Their accession numbers are specified in the phylogenetic tree (Fig. 1). The final 28S dataset included 135 sequences and resulted in an alignment of 993 characters. The ML and BI analyses resulted in nearly identical topologies, and thus only the ML tree is presented with the BS and BPP when they were greater than or equal to 50% and 0.90, respectively (Fig. 1).

The phylogeny inferred from the 28S dataset (Fig. 1) shows that the specimen JV 2106/102 together with one specimen (MUCL 45062 from Cuba) form a distinct lineage and that the specimen JV2109/73 forms another independent *Phylloporia* lineage.

### ﻿Taxonomy

#### ﻿ Phylloporia crystallina

Taxon classificationFungiHymenochaetalesHymenochaetaceae

Y.C. Dai, F. Wu, Meng Zhou & Vlasák sp. nov.

2EAC5E3B-F249-57ED-84EC-9C68089ABD5A

843482

Figs 2 , 3

##### Type.

Ecuador, Mindo Valley, San Carlos, Cascadas; alt. 1400m; 0°4'S, 78°45'W; 20 Jun. 2021; Vlasák leg.; on angiosperm freshly dead stump in tropical cloud forest; JV2106/102 (holotype BJFC038563, isotype PRM957106). GenBank: ON129551 (ITS); ON006467 (LSU)

##### Etymology.

— *Crystallina* (Lat.): refer to the species having abundant large rhomboid crystals in tube trama.

##### Diagnosis.

*Phylloporiacrystallina* is characterized by pileate, perennial basidiomata with a thin layer of context between individual tube layers, a duplex context with a black line separating the upper tomentum and a lower compacted layer, small pores 9–10 per mm, a monomitic hyphal system, generative hyphae thin- to distinctly thick-walled with simple septa, the absence of cystidia and cystidioles, the presence of large rhomboid crystals in tube trama, broadly ellipsoid basidiospores measuring 2.8–3 × 2–2.3 μm, and growth on angiosperm stump in the Neotropics.

##### Basidiomata.

Perennial, effused reflexed, imbricate, broadly attached to the substrate, hard corky when fresh, woody hard when dry. Pilei applanate to semi-circular, projecting up to 2 cm and 3 cm wide. Pileal surface curry yellow to cinnamon buff when fresh, become purplish chestnut when dry, concentrically sulcate with narrow zones, densely tomentum when juvenile, become velutinate to matted with age, the tomentum up to 1 mm thick, wearing off, leaving a dense trichoderm, sometime covered by mosses; margin sharp, entire. Pore surface pinkish buff to buff yellow and glancing when fresh, become honey yellow when dry; pores round, 9–10 per mm; dissepiments thin, entire. Context umber, up to 3 mm thick, duplex, with a black line separating the upper tomentum and a lower compacted layer, the upper tomentum soft corky, the lower layer hard corky. Tubes fulvous, paler than context, up to 5 mm long, distinctly stratified, usually filled a thin context among tube layers.

**Figure 2.**
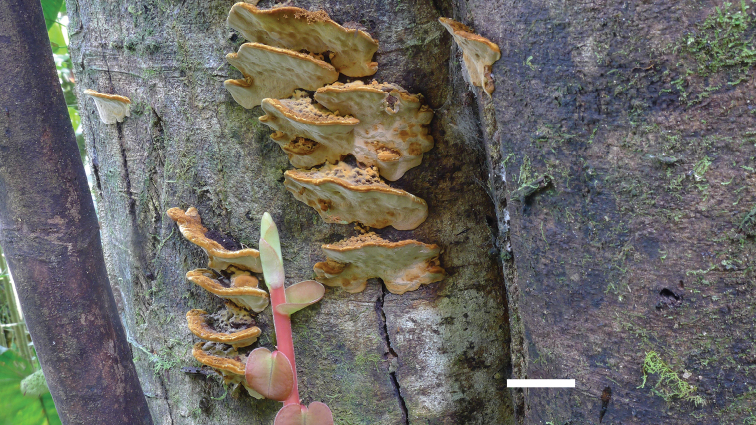
Basidiomata of *Phylloporiacrystallina* (holotype, JV2106/102). Scale bar: 1 cm.

**Figure 3.**
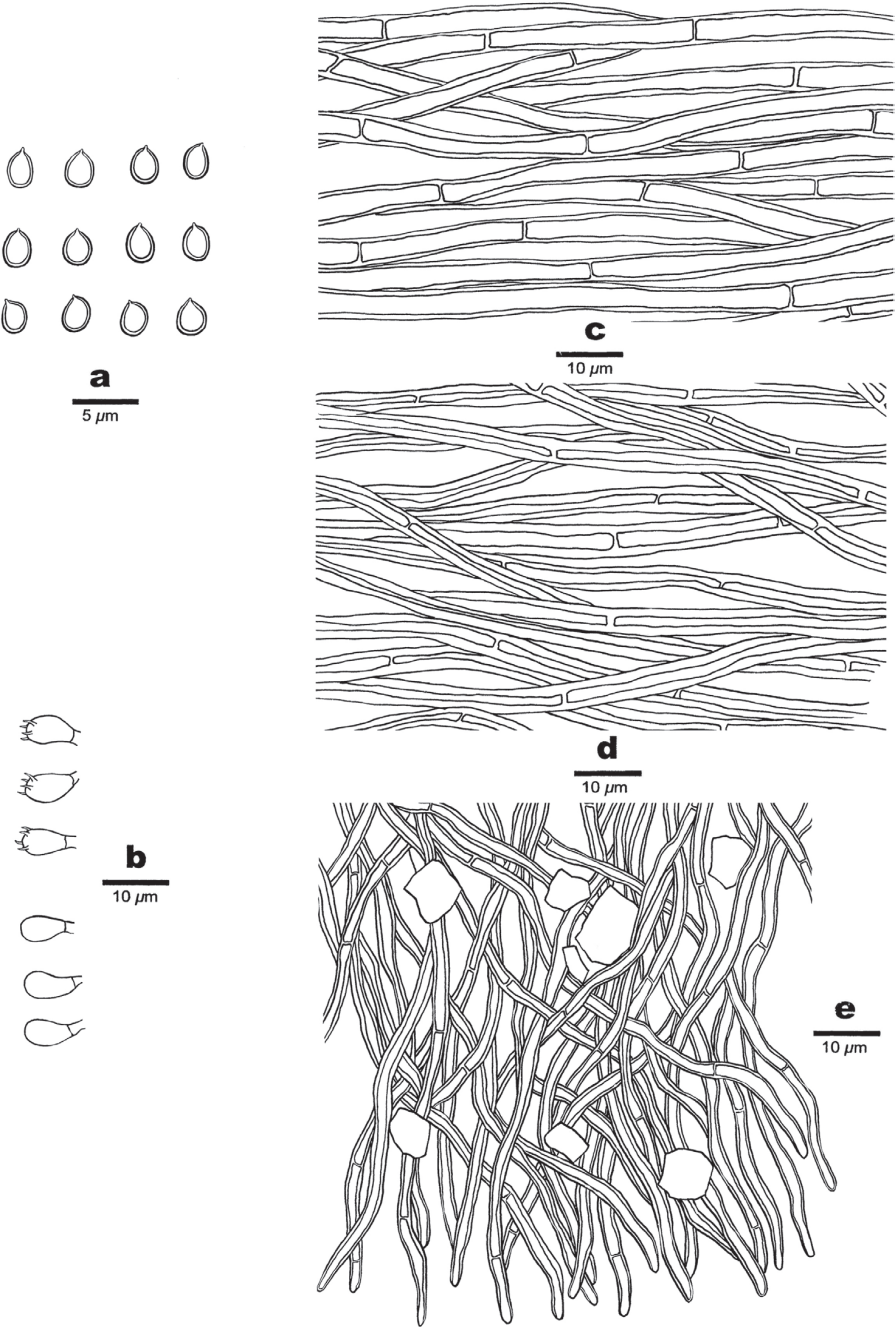
Microscopic structures of *Phylloporiacrystallina* (drawn from the holotype, JV2106/102). **a** basidiospores **b** basidia and basidioles **c** hyphae from upper tomentum **d** hyphae from lower compacted context **e** hyphae from dissepiment edge.

##### Hyphal structure.

Hyphal system monomitic; generative hyphae simple septate; tissue darkening but otherwise unchanged in the shape of the hyphae in KOH.

***Context*.** Hyphae in the lower context golden yellow, fairly thick-walled with a wide lumen, unbranched, frequently simple-septate, loosely interwoven, slightly CB+, 3–5 μm diam.; hyphae in the upper tomentum yellow, fairly thick-walled with a wide lumen, unbranched, frequently simple septate, straight, regularly arranged, 5–7 μm diam.

***Tubes*.** Tramal hyphae hyaline to yellow, thin- to thick-walled with a narrow to medium lumen, rarely branched, frequently to occasionally simple septate, flexuous, loosely interwoven, slightly CB+, 2–3.5 μm diam.; hyphae at dissepiment edges smooth; large rhomboid crystals abundant among tube trama.

***Hymenium*.** Cystidia and cystidioles absent; basidia barrel-shaped with four sterigmata and a simple septum at the base, 5–7 × 3.5–4 μm. Basidioles similar to basidia in shape, but slightly smaller. Basidiospores broadly ellipsoid to subglobose, yellowish, thick-walled, smooth, not collapsed, IKI–, CB–, (2.7–) 2.8–3 (–3.1) × 2–2.3 (–2.4) μm, L = 2.9 μm, W = 2.1 μm, Q = 1.38 (n = 30/1).

##### Notes.

Phylogenetically (Fig. 1), *Phylloporiacrystallina* is related to *P.montana* Oliveira-Filho & Gibertoni (Wu et al. 2019). However, *P.montana* has wider pores (3–5 per mm vs. 9–10 per mm) and larger and cylindrical basidiospores (4–5 × 2–3 μm vs. 2.8–3 × 2–2.3 μm) (Wu et al. 2019). Morphologically, *P.crystallina* resembles *P.crataegi* L.W. Zhou & Y.C. Dai by sharing perennial and pileate basidiomata with duplex context, a monomitic hyphal system, interwoven tramal hyphae, the absence of cystidia and cystidioles, and broadly ellipsoid to subglobose basidiospores (Zhou and Dai 2012). However, the latter species differs from *P.crystallina* by the absence of rhomboid crystals, distinctly longer basidia (8–11 μm vs. 5–7 μm), and growth on living *Crataegus* in temperate China (Zhou and Dai 2012). In addition, *P.crystallina* and *P.crataegi* are phylogenetically distantly related (Fig. 1). *P.chrysites* (Berk.) Ryvarden is a Neotropical species. It has similar basidiospores as *P.crystallina*, but the former is readily distinguished from the latter by its annual habit and larger pores (9–10 per mm vs. pores 6–8 per mm, Wu et al. 2022).

*Trameteslilliputiana* Speg. and *Pyropolyporussubpectinatus* Murrill were originally described from Brazil and Cuba, respectively (Spegazzini 1889; Murrill 1908), and they were treated as synonyms of *Phylloporiapectinata* (Klotzsch) Ryvarden (Bresadola 1912; Ryvarden 1985; Rajchenberg and Wright 1987). However, these two taxa may be different from *Phylloporiapectinata* because its type locality is in India (Wu et al. 2022). The type of *T.lilliputiana* is sterile, but its pilei are confluent and thin, and its upper surface is smooth according to its original description (Spegazzini 1889). *P.subpectinatus* has globose basidiospores (Murrill 1908). So, these two taxa are closer or identical to *P.pectinata* which has a dimitic hyphal structure and globose basidiospores (Ryvarden 2004); while *P.crystallina* has a monomitic hyphal system and broadly ellipsoid basidiospores.

#### ﻿ Phylloporia sumacoensis

Taxon classificationFungiHymenochaetalesHymenochaetaceae

Y.C. Dai, F. Wu, Meng Zhou & Vlasák sp. nov.

C58E74F9-09D6-5885-A372-835B90CFB8D1

843484

Figs 4 , 5

##### Type.

Ecuador, Guamani, Wild Sumaco Lodge; alt. 1200m; 0°40'S, 77°36'W; 30. Sep. 2021; Vlasák leg.; on living liana in tropical cloud forest; JV2109/73 (holotype BJFC038576, isotype PRM957107). GenBank: ON129552 (ITS); ON006468 (LSU).

##### Etymology.

— *Sumacoensis* (Lat.): refer to the species being found close to Sumaco Vulcan, Ecuador.

##### Diagnosis.

*Phylloporiasumacoensis* is characterized by pileate, perennial basidiomata with a thin layer of context between individual tube layers, a duplex context with a black line separating the upper tomentum and a lower compacted layer, very small pores 10–12 per mm, a monomitic hyphal system, generative hyphae thin- to distinctly thick-walled with simple septa, the hyphae at dissepiment edges bearing fine crystals, presence of cystidioles, broadly ellipsoid to subglobose basidiospores as 3–3.7 × 2.1–2.8 μm, and growth on living liana at medium elevation in the Neotropical cloud forest.

##### Basidiomata.

Perennial, pileate, solitary, broadly attached to the substrate, corky when fresh, hard corky when dry. Pilei applanate to semi-circular, projecting up to 4 cm, 5 cm wide and 15 mm thick at base. Pileal surface fuscous to vinaceous gray when fresh, become fulvous to date brown when dry, concentrically zonate and sulcate, densely tomentose, the tomentum up to 4 mm thick; margin obtuse, entire. Pore surface brownish gray to yellowish gray and glancing when fresh, become snuff brown when dry; pores round, 10–12 per mm; dissepiments thick, entire. Context fulvous, up to 8 mm thick, duplex, with a black line separating an upper soft corky tomentum, up to 4 mm thick and the lower compacted layer, hard corky, up to 4 mm thick. Tubes fawn, darker than context, up to 7 mm long, distinctly stratified, usually with a thin layer of context between individual tube layers.

**Figure 4.**
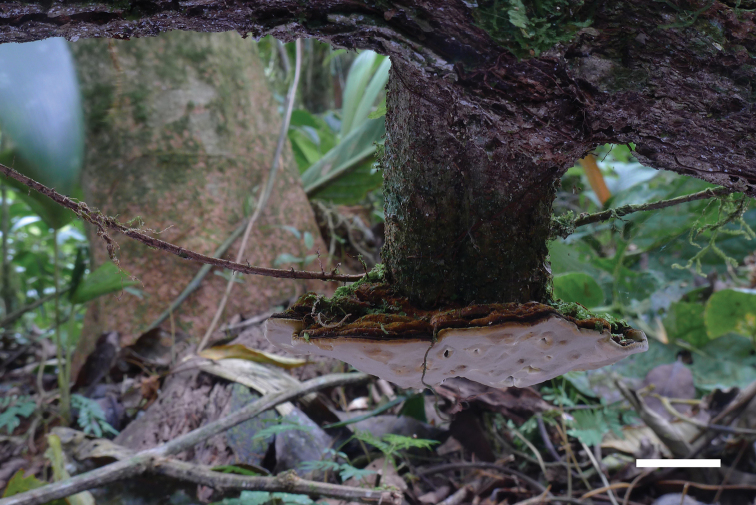
A basidiomata of *Phylloporiasumacoensis* (holotype, JV2109/73). Scale bar: 1 cm.

**Figure 5.**
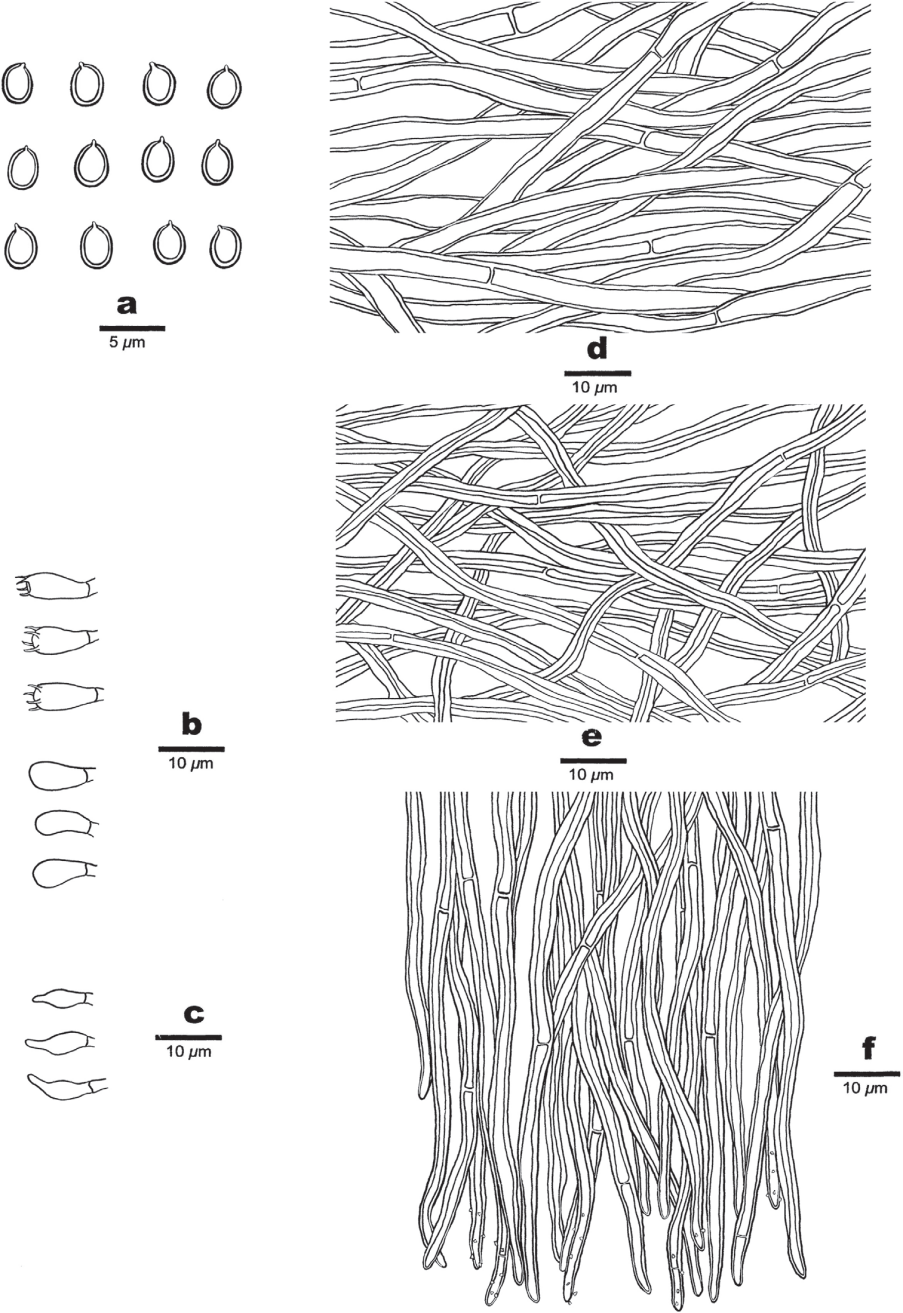
Microscopic structures of *Phylloporiasumacoensis* (drawn from the holotype, JV2109/73). **a** basidiospores **b** basidia and basidioles **c** cystidioles **d** hyphae from upper tomentum **e** hyphae from lower compacted context **f** hyphae from dissepiment edge.

##### Hyphal structure.

Hyphal system monomitic; generative hyphae simple septate; tissue darkening but otherwise unchanged in the shape of the hyphae in KOH.

***Context.*** Hyphae in the lower context golden yellow, thick-walled with a narrow to medium lumen, unbranched, occasionally simple septate, interwoven, 3–5 μm diam.; hyphae in the tomentum brownish yellow, fairly thick-walled with a wide lumen, unbranched, frequently simple septate, some collapsed, loosely interwoven, 5–7 μm diam.

***Tubes*.** Tramal hyphae hyaline to golden yellow, thin- to thick-walled with a narrow to medium lumen, rarely branched, frequently to occasionally simple septate, parallel or subparallel along the tubes, 2–4 μm diam.; hyphae at dissepiment edges bearing fine crystals.

***Hymenium*.** Cystidia absent, fusoid cystidioles rarely present; basidia barrel-shaped with four sterigmata and a simple septum at the base, 10–12 × 4.5–5 μm. Basidioles similar to basidia in shape, but slightly smaller. Basidiospores broadly ellipsoid to subglobose, yellowish, thick-walled, smooth, some collapsed, IKI–, CB–, (2.9–)3–3.7(–3.9) × 2.1–2.8 μm, L = 3.18 μm, W = 2.48 μm, Q = 1.28 (n = 30/1).

##### Notes.

Phylogenetically, *Phylloporiasumacoensis* is closely related to two other Neotropical species, *P.spathulata* (Hook.) Ryvarden sensu auctore and *P.ulloae* R. Valenz. et al. (Fig. 1). However, *P.spathulata* differs from *P.sumacoensis* in having stipitate basidiomata, wider pores (7–9 per mm vs. 10–12 per mm), and the absence of cystidioles (Ryvarden 2004). *Phylloporiaulloae* differs from *P.sumacoensis* in having wider pores (6–8 per mm vs. 10–12 per mm) and longer basidia (14.5–16 μm vs. 10–12 μm) (Valenzuela et al. 2011). Morphologically, *P.sumacoensis* is similar to *P.fontanesiae* L.W. Zhou & Y.C. Dai by sharing same pores size and broadly ellipsoid basidiospore (Zhou and Dai 2012), but the latter species has an annual habit, shorter basidia (6–7 × 3.5–4 µm vs. 10–12 × 4.5–5 μm), shorter basidiospores (2.5–3 μm vs. 3–3.7 μm), and growth on living *Fontanesia* in Asia (Zhou and Dai 2012). In addition, *P.sumacoensis* and *P.fontanesiae* are phylogenetically distantly related (Fig. 1).

## ﻿Discussion

Most *Phylloporia* species grow parasitically on living hardwoods, and speciation in the genus seems to be driven by the process of colonizing and adapting to new hosts (Wu et al. 2019). The genus is taxonomically difficult, however, because of the similar morphology among species. Before the era of molecular phylogenetics, the diversity of *Phylloporia* was grossly underestimated. The genus has received considerable attention through LSU-based phylogenetic studies (Wagner and Ryvarden 2002). So far, 73 species in the genus are accepted, and most of them were described and confirmed by molecular data recently (Wu et al. 2019, 2022). In addition, the majority of the recently described species were from subtropical and tropical areas, pointing to a remarkable species richness of the genus in tropical regions. There can be little doubt that more undescribed taxa of *Phylloporia* are present in the Neotropics, and the more samples are collected the better our understanding of species diversity of the genus will be.

Unlike other wood-inhabiting fungal genera, very long and complex ITS sequences are present in most *Phylloporia* species. These are difficult to align confidently. Accordingly, most phylogenies were based on LSU sequences (Decock et al. 2015; Yombiyeni et al. 2015; Ferreira-Lopes et al. 2016; Zhou 2016; Rajchenberg et al. 2019; Wu et al. 2019, 2022). Although *Phylloporia* is shown to be monophyletic based on LSU sequences, the genus is composed of a non-trivial number of subclades (Fig. 1). In addition, in some cases, phylogenetic estimates of *Phylloporia* look strikingly different depending on what exact taxa are included in the analyses. Phylogenetic inference based on multiple genetic markers is a better solution, but most described taxa are represented by only a very limited number of sequences and different genetic markers in GenBank. Sequences from multiple genetic markers from samples of *Phylloporia* are much needed, from fairly conserved genetic markers. Ideally, the next version of MAFFT and other multiple sequence alignment tools will furthermore be able to handle the ITS sequences of the genus *Phylloporia* in a better way.

## Supplementary Material

XML Treatment for Phylloporia crystallina

XML Treatment for Phylloporia sumacoensis

## Supplementary materials

Supplementary material 1 The sequence alignments and tree Data type NEX file Explanation note The NEX file includes Phylloporia phylogenetic sequence alignments inferred from the 28S dataset and the topology of the ML analysis. https://binary.pensoft.net/file/693158This dataset is made available under the Open Database License (http://opendatacommons.org/licenses/odbl/1.0/). The Open Database License (ODbL) is a license agreement intended to allow users to freely share, modify, and use this Dataset while maintaining this same freedom for others, provided that the original source and author(s) are credited.Meng Zhou, Fang Wu, Yu-Cheng Dai, Josef Vlasák
